# Recyclability of Flame-Retardant Polypropylene: Property and Flame Retardancy Assessment

**DOI:** 10.3390/polym18070845

**Published:** 2026-03-31

**Authors:** Giulia Bernagozzi, Rossella Arrigo, Yue Xu, Miaojun Xu, Mattia Bartoli, Alberto Frache

**Affiliations:** 1 Department of Applied Science and Technology, Politecnico di Torino, Viale Teresa Michel 5, 15121 Alessandria, Italy; giulia.bernagozzi@polito.it (G.B.); alberto.frache@polito.it (A.F.); 2 Consorzio Interuniversitario Nazionale per la Scienza e Tecnologia dei Materiali (INSTM), Via G. Giusti 9, 50121 Florence, Italy; mattia.bartoli@iit.it; 3 Heilongjiang Key Laboratory of Molecular Design and Preparation of Flame Retarded Materials, College of Chemistry, Chemical Engineering and Resource Utilization, Northeast Forestry University, Harbin 150040, China; xuyue@nefu.edu.cn (Y.X.); xumiaojun@nefu.edu.cn (M.X.); 4 Center for Sustainable Future Technologies—CSFT@POLITO, Via Livorno 60, 10144 Torino, Italy

**Keywords:** polypropylene, flame-retardant, cone calorimeter tests, reprocessing, mechanical recycling

## Abstract

In the framework of plastic circularity, managing end-of-life plastics containing flame-retardant (FR) additives represents a significant challenge. Although FRs are essential for enhancing fire safety in polymeric materials, many FR-containing products are never exposed to fire during their service life. As a result, substantial amounts of still-active FR remain in plastic waste streams. Since mechanical recycling is currently the most widely implemented strategy for plastic waste management, it is crucial to evaluate whether this process affects the flammability and combustion behavior of FR plastics. In this study, polypropylene (PP) containing 21 wt.% intumescent FR (IFR) was reprocessed up to five times to simulate mechanical recycling. After each cycle, the materials were systematically characterized in terms of rheological, morphological, combustion, and mechanical behavior. Although the agglomeration of IFR particles was observed after multiple cycles, the materials maintained stable processability and thermal stability. Importantly, the charring efficiency of the IFR system was preserved, resulting in consistent flammability performance; furthermore, all reprocessed samples achieved UL 94 V-0 classification and exhibited comparable limited oxygen index values. Mechanical properties were likewise largely maintained. Overall, these findings demonstrate that mechanical recycling represents a viable end-of-life strategy for this PP/IFR system, supporting its compatibility with circular material flow.

## 1. Introduction

In recent years, the transition toward a circular economy for plastics has stimulated growing interest in strategies capable of extending the service life of polymeric materials while reducing their environmental footprint. Mechanical recycling currently represents the most widely adopted and economically viable route for plastic waste management, as it enables the recovery of materials through relatively simple reprocessing operations without extensive chemical transformation [1]. However, repeated processing is known to affect polymer structure and performance, especially in the case of widely used thermoplastics such as polypropylene (PP), which may undergo thermo-mechanical degradation, chain scission, and changes in molecular weight distribution during successive extrusion cycles [2]. These microstructural modifications can negatively affect the mechanical properties, processability, and long-term performance of recycled plastics, thus limiting their effective reintegration into new value-added applications [2].

The presence of functional additives, such as flame retardants (FR), complicates both the recyclability and the end-of-life behavior of many polymeric systems. Flame retardants play an essential role in enhancing the fire safety of polymeric materials, which are intrinsically prone to ignition and often exhibit inadequate resistance to heat and flame propagation [3]. Their primary function is expected to manifest at the final stage of a product’s life, specifically under fire conditions, where FRs act to suppress or delay combustion. Nevertheless, a substantial amount of FR-containing plastics never encounter a fire scenario during their service life. Consequently, these additives may remain chemically active and structurally intact when the plastic components enter waste streams and undergo recycling processes [4]. Their persistence raises significant concerns regarding the recyclability and circularity of FR polymers. In fact, the presence of active FRs can alter the physico-chemical properties of recycled materials, potentially compromising their performances, processability and safety [5]. More specifically, FR may degrade during the re-processing step, promote the release of toxic gases, or contaminate the recyclate, ultimately leading to a loss of added value [4]. Moreover, the heterogeneity and limited traceability of FRs in post-consumer waste streams pose additional barriers for both mechanical and chemical recycling routes [6].

As a consequence, most plastic products containing FRs are still not effectively recycled and generally follow linear end-of-life pathways such as landfilling or incineration, posing several long-term environmental risks [3]. In fact, landfilling may result in the long-term accumulation of FR-containing materials, whereas incineration can generate hazardous by-products depending on the flame-retardant chemistry and combustion conditions [7]. Therefore, improving the recyclability of flame-retardant plastics is essential for promoting a higher degree of material circularity and reducing dependence on virgin polymers.

Despite the growing relevance of this issue, the current understanding of the behavior of flame-retardant polymers during service life and mechanical recycling remains limited. A growing number of research papers have investigated the aging behavior of flame-retarded polymer-based composites, demonstrating how thermal and thermo-oxidative aging mechanisms can affect both the polymer matrix and the efficacy of the flame retardant system, especially those incorporating intumescent flame-retardant (IFR) [8,9,10,11]. In contrast, studies specifically addressing the effect of mechanical recycling on flame retardancy are still few and relatively dated [9,12,13]. This lack of information is particularly critical because repeated processing steps may alter not only the polymer matrix but also the distribution, chemical stability, and efficiency of the flame-retardant additives. In FR-containing PP systems, the combined effect of thermo-mechanical degradation of the matrix and possible modification of the flame-retardant system may lead to significant changes in processability, mechanical performance, and also combustion behavior. For instance, degradation of the polymer or partial volatilization and redistribution of FR additives during extrusion may significantly affect ignition behavior, heat release rate, and charring efficiency. Such alterations may lead to markedly different responses under fire scenarios, diminishing the effectiveness of the original flame-retardant system [9,12]. Furthermore, the repeated exposure of FR polymers to high shear and thermal stresses during multiple reprocessing cycles can negatively impact their processability. Typical manifestations include decreased melt viscosity, reduced thermal stability, and changes in the rheological behavior, all of which may hinder the ability to manufacture high-quality recycled materials [14]. As the number of recycling cycles increases, these effects tend to accumulate, making it essential to adapt processing conditions, such as temperature profile, residence times, and screw configuration, to maintain acceptable material properties and avoid further degradation. Overall, these aspects indicate that the influence of repeated reprocessing on the properties and flame-retardant performance of FR-containing polymers is still insufficiently understood.

To address this knowledge gap, the present study investigates the influence of up to five reprocessing cycles on a PP formulation containing 21 wt.% intumescent flame-retardant system composed of piperazine pyrophosphate (PPAP) and melamine pyrophosphate (MPP), whose mixture is very effective for the flame retardancy of PP-based composites [15,16,17,18,19,20]. Their mechanism of action, which involved both condensed-phase char formation and gas-phase flame inhibition, has been clearly explained by Yuan et al. [18]. Moreover, it has already been demonstrated that MPP was able to inhibit the photoaging process of PP [7]. By simulating the conditions typically encountered in industrial mechanical recycling, this work provides a comprehensive assessment of the rheological, morphological, combustion and mechanical properties of the reprocessed material. The results aim to clarify whether intumescent FR systems can withstand repeated processing without compromising fire performance or mechanical integrity, thereby supporting the development of recycling-compatible FR formulations suitable for circular plastic streams.

## 2. Materials and Methods

### 2.1. Materials

MOPLEN HP500N polypropylene (PP) from LyondellBasell (Rotterdam, The Netherlands), with a melt flow index of 12 g/10 min (230 °C/2.16 kg), was employed as the polymer matrix.

An intumescent flame-retardant (IFR) mixture of piperazine pyrophosphate (PAPP, Zhongshan Complord New Materials Co., Ltd., Zhongshan, China) and melamine pyrophosphate (MPP, Zhenjiang Senhua Flame Retardant Engineering Technology Co., Ltd., Zhenjiang, China) in a 2:1 mass ratio was employed, following previously reported synergistic behavior [18,20,21]. The amount of IFR embedded in PP was 21 wt.%.

### 2.2. Processing

The melt processing was carried out by means of a twin-screw extruder (Leistritz (Nuremberg, Germany) ZSE 18 HP) at 900 rpm, a flow rate of 3 kg/h, and the following temperature profiles (°C): 160, 170, 180, 180, 180, and 180. All the materials were dried in an oven at 80 °C for 2 h before each processing step. Virgin PP (vPP) was melt-compounded with 21 wt.% of PAPP:MPP in a ratio equal to 2:1 (vPP + IFR), and five subsequent extrusions were conducted in order to simulate the conditions to which the plastics are subjected during a typical mechanical recycling process. Therefore, vPP + IFR was melt-compounded, collected, characterized and then reprocessed again, aiming at evaluating the variations in the properties when this kind of material reaches its end-of-life and needs to be recycled. The so-obtained materials were named, highlighting the number of reprocessing cycles (Table 1).

An injection molding machine, Haitian (Xiaogang, Beilun, Ningbo, China) HTF86X 1, was employed to produce the specimens for fire performances and mechanical characterizations. The screw temperature profile was set to 165–175–180–180 °C, and the mold was maintained at 30 °C.

### 2.3. Characterization Methods

A strain-controlled rheometer ARES (TA Instruments, Milford, MA, USA) was used for evaluating the rheological behavior of the materials investigated. All measurements were carried out under a nitrogen atmosphere. Frequency sweep tests were performed at 180 °C across a 100–0.1 rad/s frequency range, within the linear viscoelastic region (preliminarily assessed through strain sweep measurements) of each sample.

The thermal stability was evaluated through thermogravimetric analyses (TGA 8000, PerkinElmer, Waltham, MA, USA) under air atmosphere, applying a constant heating rate of 10 °C/min. The following results were obtained: T_onset_ (temperature at which 2% of weight loss occurs), T_max_ (temperature at which maximum weight loss rate is observed in dTG—derivative—curves), and the residue at 600 °C.

The limiting oxygen index (LOI) was measured on specimens of 80 × 10 × 3 mm^3^ using a JF-3 oxygen index tester (Jiangning, China), following ASTM D2863 [22]. Vertical burning performance (UL 94) was tested in accordance with ASTM D3801 [23] using a CZF-5 horizontal and vertical combustion tester (China Jiangning Analytical Instrument Co., Nanjing, China), with samples sized 130 × 13 × 3.2 mm^3^. Cone calorimeter tests (Vouch-6810, Suzhou Yangyi Walch Testing Technology Co., Jingsu, China) were conducted according to ISO 5660 at a heat flux of 50 kW/m^2^, employing samples of 100 × 100 × 4 mm^3^ to further analyze the combustion behavior. The cone calorimeter tests were carried out on three samples, and the results were averaged.

The following quantitative parameters were calculated from the cone calorimeter tests: the specific smoke extinction area (SEA), reflecting the smoke production efficiency; the effective heat of combustion (EHC), useful to evaluate the combustion efficiency of the produced gases; the maximum average rate of heat emission (MARHE), which provides information about the intensity of the flame [24,25].

A scanning electron microscope (SEM, Apreo C, Thermo Scientific, Waltham, MA, USA) was employed to analyze the morphology of each sample. The resulting images were quantitatively processed using ImageJ (1.54k (National Institutes of Health, Bethesda, MD, USA)) software, and the distribution of the Feret diameter, used to measure the dimensions of irregular and micrometric particles [26], was determined. The element composition of the residual char after the cone calorimeter test was investigated through Energy-dispersive X-ray Spectroscopy (EDX).

Raman spectroscopy was performed at ambient conditions with a DXR2 Raman Microscope (Thermo Scientific), using a 532 nm laser.

An RGT-20A universal testing machine (Reger, Shenzhen, China) was used for the mechanical characterizations. Tensile tests were carried out at a crosshead speed of 25 mm/min, while flexural tests were performed at 15 mm/min. Impact resistance was evaluated by notched Izod impact testing with a ZwickRoell (Ulm, Germany) HIT25P instrument, employing a 5.5 J pendulum impact. All the mechanical properties were carried out on five samples, and the results were averaged.

## 3. Results and Discussion

### 3.1. Thermal Stability upon Reprocessing

**Figure 1:**
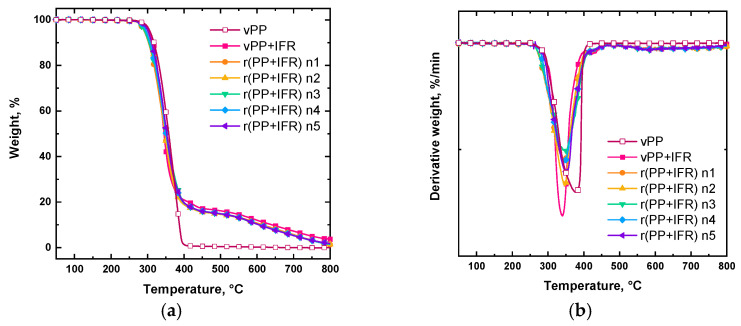
(**a**) TGA and (**b**) dTG curves of unfilled and IFR-containing virgin and reprocessed PP-based systems.

TGA tests were employed to evaluate the thermo-oxidative stability upon the introduction of IFR filler and reprocessing. The corresponding results are reported in Figure 1 and Table 2. Virgin unfilled PP started to decompose at about 294 °C with a single degradation peak at about 379 °C and zero residue at the end of the test. With the introduction of the IFR, the main thermal degradation mechanism of PP did not change. In fact, vPP + IFR started to decompose at approximately 287 °C, exhibiting the same main degradation step as unfilled PP, notwithstanding the occurrence of a second degradation step at higher temperatures, due to the IFR reactions [15,16,17,18]. In vPP + IFR, the final residue is approximately 12% at 600 °C, due to the charring ability of the mixture PAPP:MPP.

After analyzing the thermo-oxidative mechanism of the virgin PP/IFR system, evaluating its stability during reprocessing is crucial to determine whether mechanical recycling may lead to flame-retardant deterioration, and consequently, to assess whether this approach for recycling is suitable for this type of system. First of all, the stability of the IFR filler was verified by performing a TGA analysis under isothermal conditions. More specifically, IFR powders were heated to 180 °C at 10 °C/min and then kept at this temperature for 10 min. As depicted in Figure S1, the IFR filler lost less than 1.2% of its initial weight after 10 min. Therefore, IFR could be considered thermally stable during the five reprocessing steps, each lasting approximately 1 min and carried out at a maximum temperature of 180 °C.

The reprocessed materials exhibited a behavior comparable to that of their virgin counterparts. In fact, the TGA curves of all reprocessed samples essentially overlapped. Compared with vPP + IFR, the one-time reprocessed material (r(PP + IFR) n1) showed a slight decrease in the onset temperature, a minor increase in the temperature of maximum degradation rate, and a marginally lower residue at 600 °C. Beyond the first reprocessing step, however, the onset temperature, peak degradation temperature, and char residue at 600 °C remained nearly constant up to the fifth cycle. Overall, the reprocessing did not remarkably affect the thermo-oxidative stability of PP/IFR systems or the charring ability of the IFR additive, at least up to five reprocessing cycles.

### 3.2. Effects of Reprocessing on PP/IFR Microstructure

**Figure 2:**
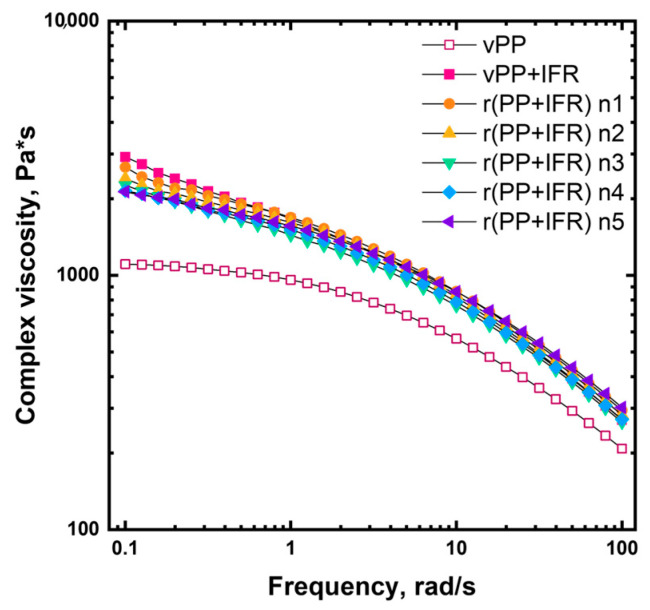
Complex viscosity curves as a function of the frequency of unfilled vPP, vPP + IFR and reprocessed samples.

Assessing the evolution of the rheological behavior of a polymeric system when subjected to reprocessing is of paramount importance when dealing with a mechanical recycling process. In fact, variations in the processability of recycled materials can pose some issues in further reprocessing since some adjustments in the processing parameters could be required. The complex viscosity curves of unfilled vPP, vPP + IFR and the five reprocessed samples are reported in Figure 2. Virgin unfilled PP showed the typical Newtonian plateau at low and intermediate frequencies, followed by a mild shear thinning as the frequencies increased. The incorporation of IFR within vPP resulted in increased complex viscosity across the investigated frequency range. More specifically, the Newtonian plateau was no longer fully visible, and a yield stress behavior appeared at low frequencies. Both these features can be ascribed to the embedded solid fillers able to hamper the mobility of the macromolecules, causing a retardation of the chain relaxation [27].

Regarding the rheological behavior of reprocessed PP/IFR systems, all the curves almost overlap at the intermediate and high frequencies, irrespective of the number of reprocessing cycles. As already reported by other authors [28,29], the reprocessing had a less marked influence on the rheological behavior of polymeric composites than on unfilled polymers. Nevertheless, in the low-frequency region, a progressive decrease in both complex viscosity and yield stress can be observed as the number of reprocessing cycles increases. This behavior can be associated with some evolution of the dispersion and distribution of the IFR within the PP matrix during reprocessing.

Furthermore, it should be pointed out that no adjustments of the processing parameters were required during the extrusions, meaning that the processability was not affected by the consecutive extrusions and all materials, starting from the virgin PP-based systems to its counterpart reprocessed five times, could be melt-compounded under the identical conditions.

**Figure 3:**
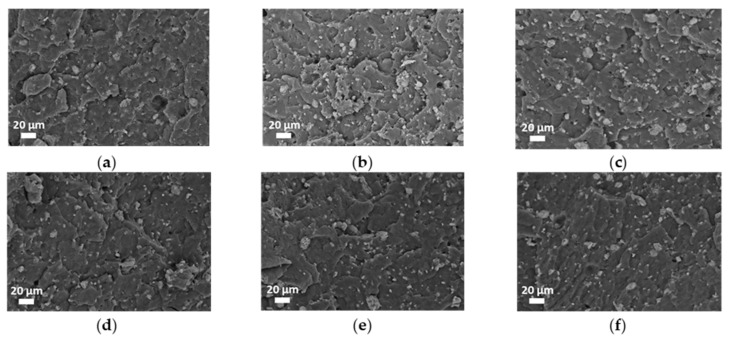
SEM micrographs of vPP + IFR (**a**) and reprocessed samples: n1 (**b**), n2 (**c**), n3 (**d**), n4 (**e**) and n5 (**f**).

**Figure 4:**
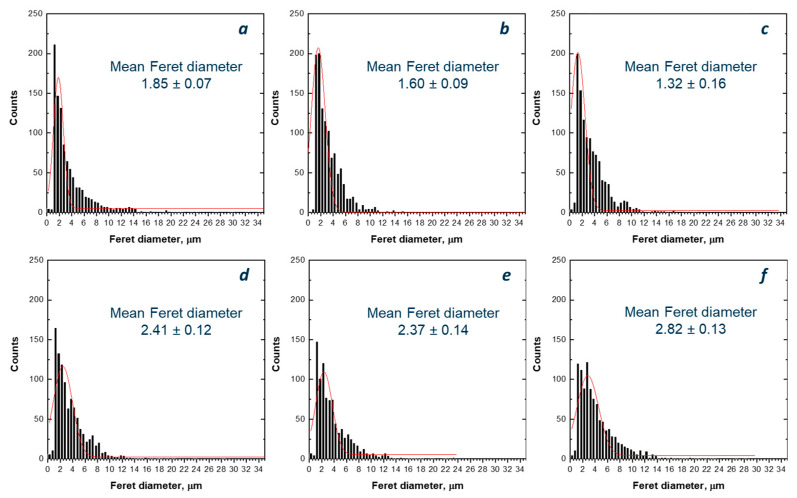
Distribution of IFR particles’ Feret diameter and mean values of vPP + IFR (**a**) and reprocessed samples: n1 (**b**), n2 (**c**), n3 (**d**), n4 (**e**) and n5 (**f**).

To assess the dispersion and distribution of IFR within the host matrix, SEM characterization was performed, and representative micrographs are reported in Figure 3. In addition, the Feret diameter distribution was evaluated for each flame-retarded system, and the obtained results are depicted in Figure 4. It is clearly observable that the reprocessing caused significant variations in particle size and dispersion. Moving from vPP + IFR (Figure 4a) to the sample reprocessed twice (Figure 4b,c), no significant differences in particle size distribution were observed, notwithstanding a slight reduction in the mean size. This observation suggests that the first two reprocessing cycles had a limited effect on IFR dispersion. Conversely, increasing the number of reprocessing steps from three to five cycles (Figure 4d–f) resulted in a progressive shift in particle size distribution towards larger diameters, accompanied by a growing fraction of coarse particles. The higher mean particle size observed after the third, fourth and fifth cycles indicates the occurrence of partial re-agglomeration of IFR particles upon repeated reprocessing.

### 3.3. Effects of Reprocessing on Flame Retardancy

The flammability of the IFR-containing systems was evaluated through UL 94 vertical burning tests, and the corresponding results for virgin and reprocessed PP/IFR materials are reported in Table 3. Virgin unfilled PP achieved no rating in the UL 94 test due to the severe dripping and burning time exceeding 30 s. Upon the introduction of IFR, the resulting material achieved a V-0 classification: samples self-extinguished after both the first and the second flame application, without producing flaming drips capable of igniting the cotton. As expected, the LOI value (reported in Table 3) also increased when IFR was introduced in vPP.

Interestingly, all the other investigated materials, irrespective of the reprocessing cycle, displayed a V-0 classification. Despite a slight increase in the burning time after the second flame application, each reprocessed sample was able to stop burning and to avoid dripping. On the other hand, LOI values remained basically unchanged, passing from the virgin FR system to the five-time reprocessed material. Therefore, the reprocessing did not affect the flammability of the system. The intumescent flame-retardant used in this study did not lose its protective properties and its ability to create a char capable of extinguishing the flame, unlike other studies in which the UL 94 classification of the materials changed from V-0 to no rating after the second reprocessing cycle [8,11] or LOI values decreased [8,11,12].

**Figure 5:**
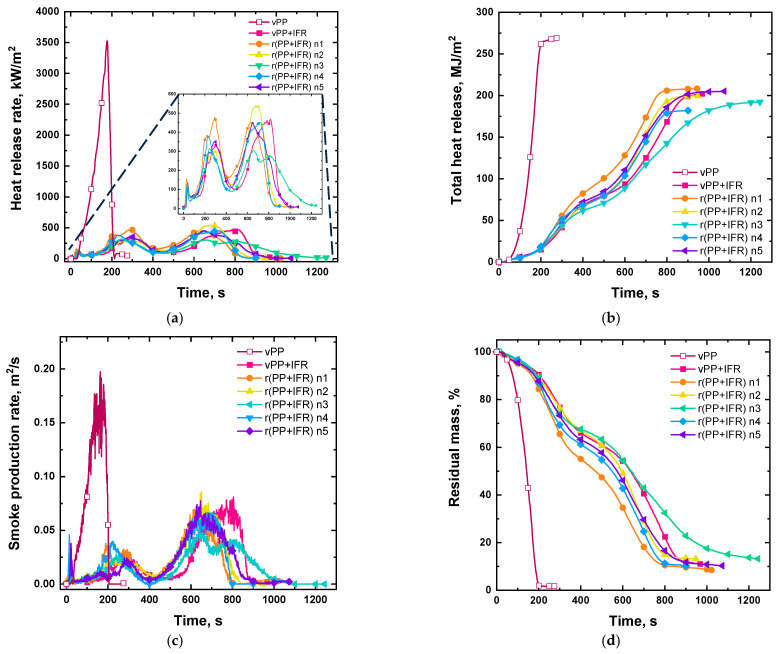
Cone calorimeter results: Heat release rate (**a**), total heat release (**b**), smoke production rate (**c**) and residual mass (**d**).

Regarding the combustion behavior, the results obtained from cone calorimeter tests are reported in Table 4 and Figure 5 (the values related to the smoke production are reported in Table S1). Without incorporating any flame-retardant, virgin PP ignited after about 34 s and burned very quickly, reaching almost immediately the heat release rate (HRR) peak and then, after consuming the sample completely, stopped burning in about 190 s. Also, the sample mass was consumed almost immediately, and the total heat was released instantly and reached the maximum in 190 s.

As already mentioned, the introduction of the mixture PAPP:MPP into virgin PP led to the development of an intumescent structure under cone calorimeter conditions. In fact, the typical HRR curve of char-forming material with two main peaks can be recognized [11,30]. The first peak can be attributed to ignition and flame spread on the surface. This is followed by a plateau effect because of the formation of a protective char layer. The second peak, which appears at a longer time, is associated with rupture of the char layer caused by volatile gases escaping from the degradation of the substrate. This explained the slower mass decrease during the tests, when compared to unfilled virgin PP, and a final solid residue consistent with TGA analysis.

Specifically, as reported in the literature [18], the charring mechanism of PAPP and MPP begins with the thermal decomposition of P–OH groups and –NH^+^_2_–OP– moieties in PAPP, accompanied by the release of H_2_O and the formation of P–O–P and P–N–C structures. With increasing temperature, MPP reacts with the residual P–OH groups in PAPP, leading to the formation of melamine salts with branched or cross-linked architectures. At higher temperatures, both the initially formed P–N–C structures and the melamine salts further decompose, producing a compact, intumescent, and thermally stable char layer, together with the release of non-flammable gases and phosphorus-containing species.

Besides the charring ability of IFR into vPP, the heat release rate decreased by about 87% for the FR system, whereas the time to ignition (TTI) was anticipated, because the addition of IFR caused early degradation phenomena of the polymeric matrix [21]. Concerning the total heat release (THR), the presence of IFR resulted in a slow increase over time, and the maximum THR value decreased by about 24% when compared to unfilled virgin PP.

What immediately emerged from the cone calorimeter graphs shown in Figure 5 was that the variations caused by reprocessing are truly minimal. In fact, irrespective of the reprocessing cycle, all the HRR curves displayed the same two main peaks as virgin PP-based materials, indicating that all the reprocessed samples are able to form the intumescent protective layer approximately at the same time of vPP + IFR after the ignition, without any significant changes in the peak values of HRR. Then, just like their virgin counterpart, for all the reprocessed samples, the second peak was ascribed to the degradation and breaking of the char layer, also in this case without any shift in the time at which the phenomena occur and with very few differences in the values of HRR peaks. There were also no significant variations in the final residual weight of the char obtained from combustion and in the time between ignition and flameout. Each cone calorimeter test performed on FR virgin and reprocessed materials proceeded in the same manner. In fact, even the weight loss of the sample proceeded in stages, and the mass loss rate (MLR) was comparable regardless of the reprocessing cycle. The time to ignition (TTI) was slightly increased as the number of recycling cycles increased, without, however, reaching the value of virgin unfilled PP.

Finally, SEA, EHC and MARHE, determined on the basis of the cone calorimeter tests, are also reported in Table 4. Since SEA remained unchanged during the reprocessing, when compared to vPP + IFR, there was no increase in smoke production for the reprocessed samples; therefore, the safety related to the smoke release was not compromised. No variations in the inhibition effects in the gas-phase combustion process were observed; indeed, the EHC values remained constant between the virgin PP-based material and the five-time reprocessed sample. In conclusion, MARHE values also remained unchanged as reprocessing occurred, meaning that the recycling did not affect fire safety or fire growth.

From the flame retardancy point of view, the composite based on virgin PP filled with 21 wt.% of the mixture PAPP:MPP could be mechanically recycled up to five times without losing the ability to be a safe material in a fire-risk scenario.

To complete the assessment of combustion behavior after reprocessing and to evaluate the suitability of these materials for fire-risk scenarios following mechanical recycling, the three key parameters describing fire intensity, fire hazard, and flame-retardant efficiency are summarized in Table 5. More specifically, the fire performance index (FPI = TTI/pkHRR), the fire growth rate index (FIGRA = pkHRR/t_pk_HRR) and the flame retardancy index (FRI) [31] were calculated. The higher the FPI value, the lower the intensity of the fire, whereas lower values of FIGRA are required for a lower fire hazard. Therefore, polymer-based systems that usually exhibit a high FPI and a low FIGRA imply great fire safety [15]. In fact, virgin PP without any flame-retardant fillers displayed very high values of FIGRA and low values of FPI. As expected, the introduction of IFR resulted in increased FPI and decreased FIGRA. Despite very small variations, none of the five reprocessing cycles changed these values. Therefore, the mechanical recycling did not worsen the fire intensity or the fire hazard; indeed, neither FPI nor FIGRA reached the original value of unfilled PP. Regarding FRI, which is a dimensionless index able to quantify the flame retardancy ability of the FR system, all the values from the virgin PP-based composite to the material reprocessed five times lie in a range between 10^0^ and 10^1^. This is a very interesting result since the values remaining in this range meant good flame retardancy performances [31].

The studied PP/IFR could then be considered safe after five reprocessing steps, thus after five rounds of mechanical recycling, also from the point of view of fire intensity and hazard.

The protective char layer formed after the combustion of the tested FR systems was further characterized in order to evaluate the charring ability, the morphology and the degree of structural evolution during reprocessing.

**Figure 6:**
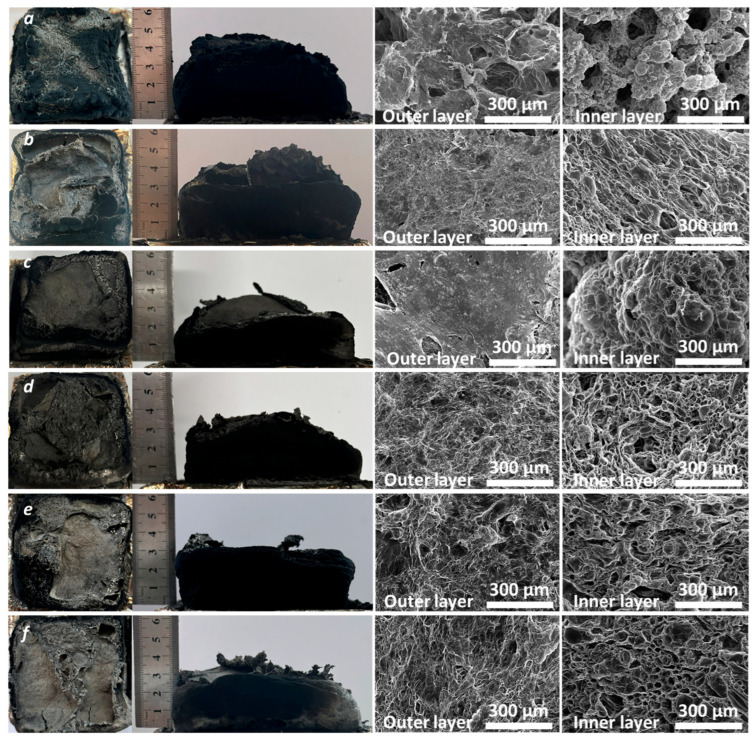
Photos and SEM images of residual char of vPP + IFR (**a**) and reprocessed samples: n1 (**b**), n2 (**c**), n3 (**d**), n4 (**e**) and n5 (**f**).

Digital images of the char residue at the end of the cone calorimeter tests (Figure 6) clearly demonstrate the charring ability of the PAPP:MPP system in PP. The char obtained from vPP + IFR (Figure 6a) exhibited a compact external surface with few holes, attributable to the gases released during MPP decomposition [21], and reached a final height of nearly 5 cm. With increasing reprocessing cycles (Figure 6b–f), the number of surface holes gradually increases, and the char height slightly decreases, remaining between 3 and 5 cm regardless of the number of cycles. These observations confirm that the overall charring ability of the IFR system in PP was not significantly affected by the reprocessing, in agreement with cone calorimeter results.

SEM analysis of the outer and inner char layers (Figure 6) revealed a distinct layered morphology for vPP + IFR: a dense, relatively intact outer surface with limited voids, and a highly porous inner structure characterized by well-defined micro-channels. After the first reprocessing cycle, the outer layer remained comparable to that of virgin PP/IFR, while the inner region exhibited smaller pores and channels. A similar morphology was observed after the second cycle. From the third to the fifth cycle, however, gradual changes became evident: the outer layers progressively lost compactness, resembling the more porous inner structure. Nevertheless, EDX analysis of the inner layers (Figure S2) confirmed a homogeneous distribution of phosphorus and nitrogen across all samples, indicating that the IFR components remained well distributed despite repeated processing.

**Figure 7:**
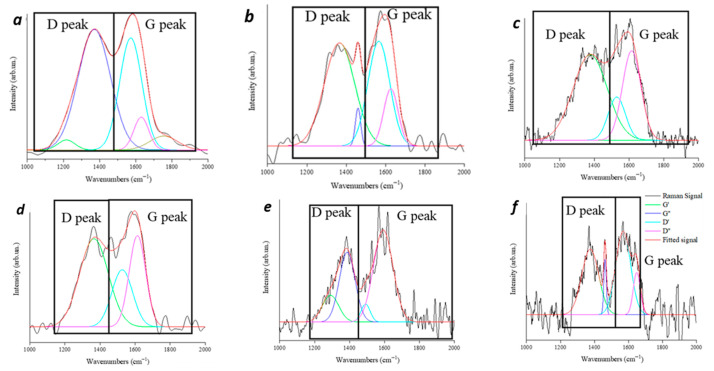
Fitted Raman spectra of residual char of vPP + IFR (**a**) and reprocessed samples: n1 (**b**), n2 (**c**), n3 (**d**), n4 (**e**) and n5 (**f**), in the region from 1000 up to 2000 cm^−1^.

The microstructure of the residual chars was further investigated through Raman analyses, and the collected spectra, showing overlapped D and G peaks [32,33], are reported in Figure 7. Furthermore, in Table 6, the ratio between the area of the two peaks (I_D_/I_G_) is listed, along with the calculated crystalline size (L_a_) [34].

The char collected from vPP + IFR exhibits a high I_D_/I_G_ up to 1.2 with a small L_a_ up to 15.9 nm, and these characteristics remain almost unchanged for the samples obtained from the first and the second reprocessing cycles. However, from the third to the fifth cycles, the I_D_/I_G_ values tend to remarkably decrease, with a significant increment in L_a_, indicating a more efficient graphitization of the material.

### 3.4. Mechanical Properties

**Figure 8:**
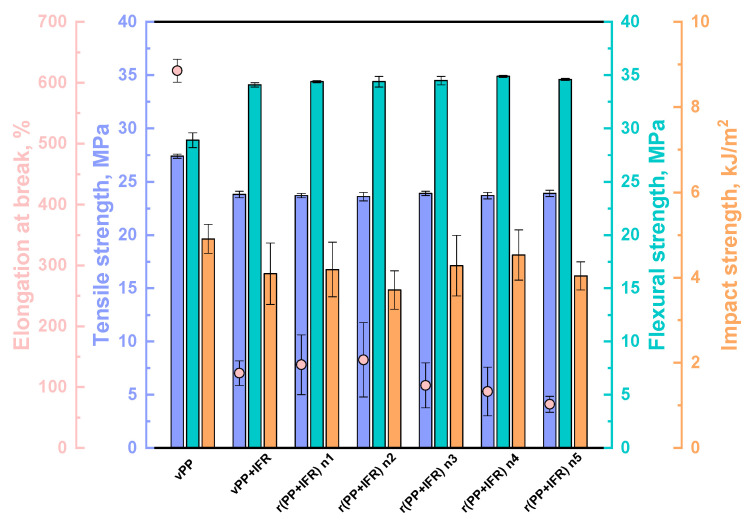
Mechanical properties of unfilled vPP, vPP + IFR and reprocessed samples.

Finally, having verified that the PP/IFR system was able to maintain its flammability and combustion properties from the virgin sample to its five-times reprocessed counterpart, tensile, flexural, and impact tests were performed in order to assess the effect of reprocessing also on the mechanical properties. The obtained results are depicted in Figure 8. First of all, the addition of IFR resulted in decreased tensile strength, elongation at break and impact strength, as compared to unfilled PP, which is also observable in the stress–strain curves reported in Figure S3. This behavior is in agreement with the literature [7,17], which already reported the poor compatibility between flame-retardants and PP as one of the main issues related to the dramatic decrease in the mechanical properties, resulting in a more brittle behavior of the FR materials. However, despite the noticeable decrease in the elongation at break with the introduction of IFR particles, the obtained values of approximately 120% were quite higher than those typically reported for PP-based composites [7,8,17,35]. Regarding the flexural strength, the mean values increased in vPP + IFR if compared to unfilled virgin PP, as already observed by other authors [36].

Very interesting results were obtained when analyzing the mechanical response upon reprocessing. Overall, tensile strength, flexural strength and impact strength were not affected by the reprocessing, keeping constant values from the virgin material to the sample reprocessed up to five times. As already shown in other works [8,11,12,37], the mechanical recycling did not induce noteworthy variations in the mechanical properties. On the other hand, the ductility was little affected by the reprocessing: elongation at break values increased slightly until the second recycling cycle, likely because of the smaller IFR particle diameter, and then gradually decreased. At the end of the fifth cycle, elongation at break decreased by about 40% if compared to the virgin PP-based system. However, all the obtained values for reprocessed samples remained higher when compared to those reported in the literature for FR virgin PP-based systems, confirming the potential of the studied materials even after mechanical recycling [7,8,17].

## 4. Conclusions

The design of plastic products frequently fails to consider their potential for reuse, repair or recycling, but this approach is no longer viable in the context of the required transition toward a plastic circular economy. This work demonstrated that flame-retardant PP-based composite with 21 wt.% of piperazine pyrophosphate and melamine pyrophosphate mixture (in a ratio 2:1) exhibited a remarkable stability under repeated mechanical recycling conditions. Despite minor morphological changes, such as the progressive agglomeration of IFR particles, neither the thermal stability nor the charring ability of the intumescent formulation was significantly affected by up to five reprocessing cycles. As a consequence, both flammability and combustion performances remained essentially unchanged, with all samples maintaining V-0 classification in UL 94 tests and comparable LOI values as compared to the virgin material. Mechanical properties were also largely preserved, aside from a slight decrease in elongation at break at higher reprocessing cycles due to cumulative thermo-mechanical degradation.

Overall, these findings indicated that mechanical recycling can be considered a viable end-of-life strategy for these PP/IFR composites, supporting their potential integration into circular plastic streams without compromising fire safety requirements.

## Figures and Tables

**Table 1:** Sample coding.

Sample Code	Processing Step	Number of Reprocessing Cycles
vPP + IFR	Melt-compounding	0
r(PP + IFR) n1	1st reprocessing	1
r(PP + IFR) n2	2nd reprocessing	2
r(PP + IFR) n3	3rd reprocessing	3
r(PP + IFR) n4	4th reprocessing	4
r(PP + IFR) n5	5th reprocessing	5

**Table 2:** Thermo-oxidative degradation data for all investigated materials.

	T_onset_ [°C]	T_max_ [°C]	Residue @600 °C [%]
vPP	294	379	0.2
vPP + IFR	287	340	12.0
r(PP + IFR) n1	280	347	10.7
r(PP + IFR) n2	287	347	10.3
r(PP + IFR) n3	280	348	10.3
r(PP + IFR) n4	286	347	10.3
r(PP + IFR) n5	288	351	10.3

**Table 3:** UL 94 vertical burning and LOI results.

	Rating	t1 [s]	t2 [s]	Dripping	LOI [%]
vPP	NR	5.4 ± 2.7	>30	Yes	21
vPP + IFR	V-0	0	3.5 ± 1.0	No	32.5
r(PP + IFR) n1	V-0	0	5.1 ± 1.6	No	33.4
r(PP + IFR) n2	V-0	0	4.6 ± 1.4	No	33.2
r(PP + IFR) n3	V-0	0	5.0 ± 2.1	No	33.8
r(PP + IFR) n4	V-0	0	5.2 ± 2.4	No	32.8
r(PP + IFR) n5	V-0	0	7.4 ± 2.2	No	32.5

**Table 4:** Results from cone calorimeter tests.

	vPP	vPP + IFR	r(PP + IFR) n1	r(PP + IFR) n2	r(PP + IFR) n3	r(PP + IFR) n4	r(PP + IFR) n5
TTI [s]	34 ± 5	13 ± 2	14 ± 3	16 ± 3	19 ± 1	21 ± 5	18 ± 7
Ignition to flameout [s]	189 ± 10	933 ± 153	958 ± 63	847 ± 97	1111 ± 166	989 ± 125	939 ± 39
peak1-HRR [kW/m^2^]	3406 ± 194	338 ± 35	428 ± 60	346 ± 56	344 ± 64	338 ± 37	372 ± 28
t peak1-HRR [s]	178 ± 9	308 ± 8	267 ± 22	303 ± 25	259 ± 15	261 ± 28	284 ± 17
peak2-HRR [kW/m^2^]	-	451 ± 17	431 ± 113	526 ± 71	315 ± 11	404 ± 60	450 ± 1
t peak2-HRR [s]	-	819 ± 126	654 ± 63	697 ± 128	618 ± 73	843 ± 127	630 ± 31
THR [MJ/m^2^]	263 ± 6	200 ± 2	206 ± 10	203 ± 13	204 ± 10	194 ± 13	197 ± 12
Char residue [wt.%]	1.2 ± 0.5	11.3 ± 0.4	9.9 ± 1.3	13.3 ± 3.5	11.1 ± 1.8	8.1 ± 0.7	9.7 ± 0.8
SEA [m^2^/kg]	651 ± 8	604 ± 27	554 ± 74	607 ± 92	620 ± 14	548 ± 36	577 ± 86
EHC [MJ/kg]	55.1 ± 3.2	48.1 ± 3.2	44.5 ± 5.8	45.4 ± 4.1	44.9 ± 5.8	48.1 ± 1.8	49.3 ± 0.7
MARHE [kW/m^2^]	1264 ± 73	220 ± 32	252 ± 27	242 ± 13	203 ± 26	201 ± 23	232 ± 10
MLR [g/s]	0.140 ± 0.007	0.030 ± 0.005	0.029 ± 0.002	0.032 ± 0.002	0.025 ± 0.004	0.030 ± 0.007	0.030 ± 0.001

**Table 5:** FPI, FIGRA and FRI values.

	FPI [m^2^ × s/kW]	FIGRA [kW/m^2^/s]	FRI
vPP	0.010 ± 0.003	19.2 ± 2.0	-
vPP + IFR	0.040 ± 0.005	1.10 ± 0.13	5.1
r(PP + IFR) n1	0.033 ± 0.012	1.60 ± 0.13	4.0
r(PP + IFR) n2	0.045 ± 0.003	1.15 ± 0.25	5.8
r(PP + IFR) n3	0.056 ± 0.011	1.32 ± 0.16	6.9
r(PP + IFR) n4	0.062 ± 0.020	1.32 ± 0.29	8.2
r(PP + IFR) n5	0.048 ± 0.028	1.31 ± 0.18	6.2

**Table 6:** I_D_/I_G_ and L_a_ of vPP + IFR and reprocessed samples: n1–n5.

Sample	I_D_/I_G_	L_a_ (nm)
vPP + IFR	1.2	15.9
r(PP + IFR) n1	1.1	17.5
r(PP + IFR) n2	1.1	17.5
r(PP + IFR) n3	0.9	21.4
r(PP + IFR) n4	0.8	24.0
r(PP + IFR) n5	0.7	27.9

## Data Availability

The original contributions presented in this study are included in the article/Supplementary Materials. Further inquiries can be directed to the corresponding author.

## Supplementary Materials

The following supporting information can be downloaded at: https://www.mdpi.com/article/10.3390/polym18070845/s1, Figure S1. Isothermal TGA at 180 °C of IFR; Figure S2. Phosphorus (top row) and nitrogen (bottom row) EDX analysis of inner char layer of vPP + IFR (a) and reprocessed samples: n1 (b), n2 (c), n3 (d), n4 (e) and n5 (f); Figure S3. Stress–strain curves for all investigated materials; Table S1: Smoke production values.
