# Supplementary material for: Recyclability of Flame-Retardant Polypropylene: Property and Flame Retardancy Assessment
Source: Polymers (Basel). 2026 Mar 31;18(7):845. doi: 10.3390/polym18070845 (PMC13074281; doi:10.3390/polym18070845)
Supplement: Supplementary file 1 [file polymers-18-00845-s001.zip › polymers-4213352-supplementary.pdf]

polymers

MDPI

Article

Recyclability of Flame-Retardant Polypropylene: Property and Flame Retardancy Assessment

Giulia Bernagozzi[1,2], Rossella Arrigo[1,2,*], Yue Xu[3], Miaojun Xu[3], Mattia Bartoli[2,4] and Alberto Frache[1,2]

[1] Department of Applied Science and Technology, Politecnico di Torino, Viale Teresa Michel 5, 15121, Alessandria, Italy; giulia.bernagozzi@polito.it, rossella.arrigo@polito.it, alberto.frache@polito.it
[2] Consorzio Interuniversitario Nazionale per la Scienza e Tecnologia dei Materiali (INSTM), Via G. Giusti 9, 50121 Florence, Italy
[3] Heilongjiang Key Laboratory of Molecular Design and Preparation of Flame Retarded Materials, College of Chemistry, Chemical Engineering and Resource Utilization, Northeast Forestry University, 150040, Harbin, China; xuyue@nefu.edu.cn, xumiaojun@nefu.edu.cn
[4] Center for Sustainable Future Technologies—CSFT@POLITO, Via Livorno 60, 10144 Torino, Italy; mattia.bartoli@iit.it (M.B.)

* Correspondence: rossella.arrigo@polito.it

Supplementary materials

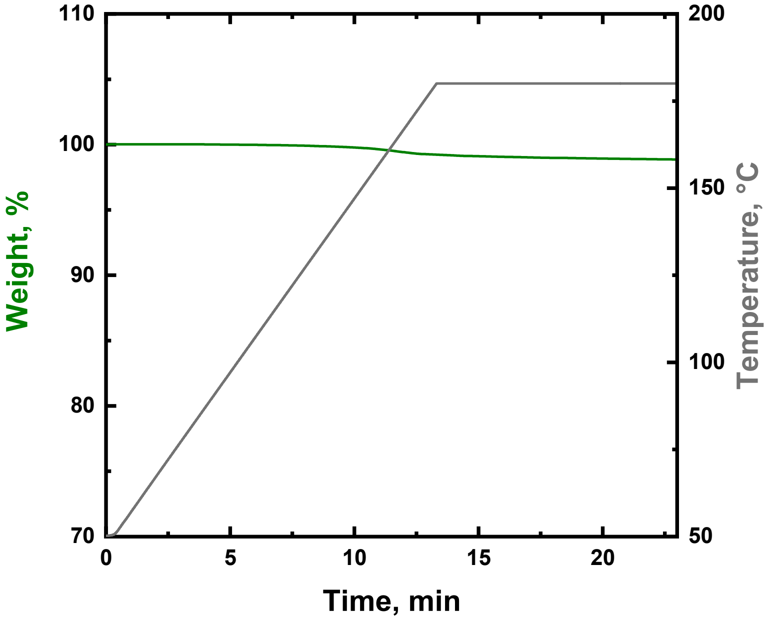

Figure S1. Isothermal TGA at 180°C of IFR.

Academic Editor: Firstname Lastname

Received: date
Revised: date
Accepted: date
Published: date

Copyright: © 2026 by the authors. Submitted for possible open access publication under the terms and conditions of the Creative Commons Attribution (CC BY) license.

Table S1. Smoke production values.

	vPP	vPP+IFR	r(PP+IFR) n1	r(PP+IFR) n2	r(PP+IFR) n3	r(PP+IFR) n4	r(PP+IFR) n5
peak1-SPR [m^2/s]	0.191 ± 0.011	0.023 ± 0.005	0.034 ± 0.07	0.026 ± 0.003	0.028 ± 0.001	0.028 ± 0.010	0.033 ± 0.014
t peak1-SPR [s]	167 ± 6	297 ± 25	228 ± 34	267 ± 24	266 ± 23	261 ± 38	260 ± 45
peak2-SPR [m^2/s]	-	0.067 ± 0.013	0.069 ± 0.025	0.079 ± 0.015	0.050 ± 0.004	0.058 ± 0.010	0.077 ± 0.001
t peak2-SPR [s]	-	801 ± 137	662 ± 48	688 ± 109	599 ± 63	824 ± 117	619 ± 38
TSP [m^2]	17.1 ± 0.5	19.2 ± 2.8	18.9 ± 1.7	18.8 ± 3.6	18.7 ± 1.6	18.0 ± 0.5	19.1 ± 0.6
TSR [m^2/m^2]	2108 ± 58	2352 ± 334	2338 ± 210	2315 ± 341	2296 ± 190	2218 ± 63	2349 ± 57

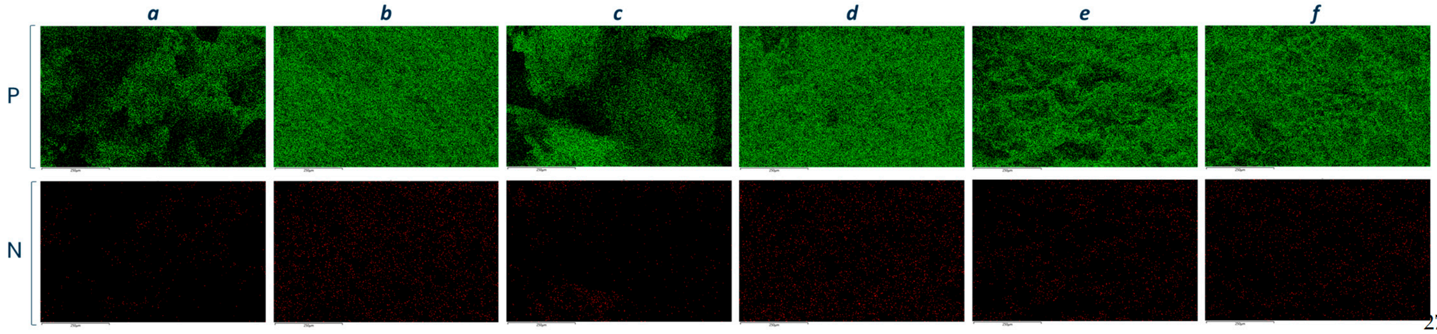

Figure S2. Phosphorus (top row) and nitrogen (bottom row) EDX analysis of inner char layer of vPP+IFR (a) and reprocessed samples: n1 (b), n2 (c), n3 (d), n4 (e) and n5 (f).

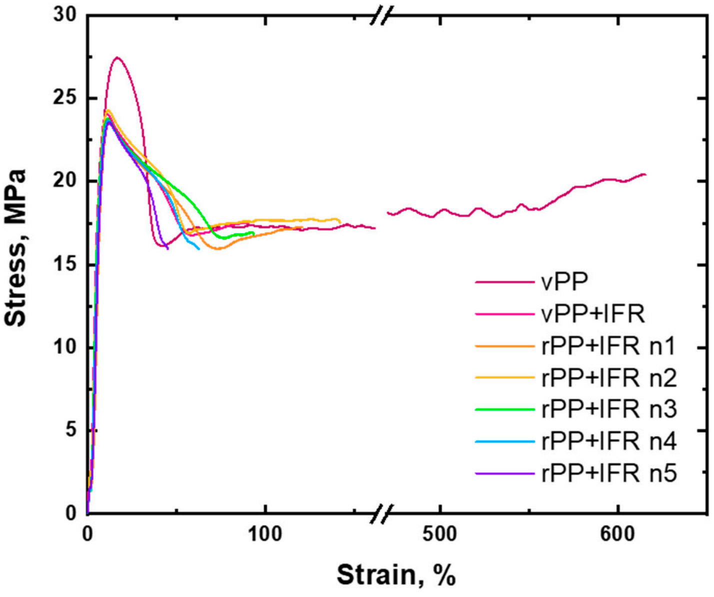

Figure S3. Stress-strain curves for all investigated materials.
